# Effects of Plant-Based Antioxidants in Animal Diets and Meat Products: A Review

**DOI:** 10.3390/foods12061334

**Published:** 2023-03-21

**Authors:** Carmen Daniela Petcu, Oana Diana Mihai, Dana Tăpăloagă, Raluca-Aniela Gheorghe-Irimia, Elena Narcisa Pogurschi, Manuella Militaru, Cristin Borda, Oana-Mărgărita Ghimpețeanu

**Affiliations:** 1 Faculty of Veterinary Medicine, University of Agronomic Sciences and Veterinary Medicine of Bucharest, 105 Blvd., Splaiul Independentei, 050097 Bucharest, Romania; 2 Faculty of Animal Productions Engineering and Management, University of Agronomic Sciences and Veterinary Medicine Bucharest, 59 Blvd., Marasti, 011464 Bucharest, Romania; 3 Faculty of Veterinary Medicine, University of Agricultural Sciences and Veterinary Medicine of Cluj-Napoca, 3-5 Mânăștur St., 400372 Cluj-Napoca, Romania

**Keywords:** natural antioxidants, food safety, meat products, risk, public health

## Abstract

The perceived level of risk associated with a food product can influence purchase and consumption decisions. Thus, current trends in food safety address an issue of general interest—the identification of healthy and economical alternatives to synthetic antioxidants that may have harmful effects on human health. Still, the processors’ target is to increase the shelf life of food products using preserving substances. Natural antioxidants can be extracted and used in the food industry from different plants, such as blueberry, broccoli, chokeberry, cinnamon, ginger, olives, oregano, etc. The identification of the main natural antioxidant types that have been used in the food industry is very important in order to provide a comprehensive analysis of the researched topic. In this regard, the aim of this paper was to illustrate the positive aspects of using natural antioxidants with preservative roles in meat products, while, at the same time, highlighting the potential risks induced by these compounds. All of those aspects are correlated with the impact of sensorial attributes and the improvement of the nutritional value of meat products.

## 1. Introduction

Food preservation was and remains a major objective of food security, aiming at the physical and economic access of all people to the basic food they need [1]. In the last decades, progress has been made regarding the identification of substances with a preservative role, increasing the shelf life of food to very long periods. However, at the same time, the use of these potentially preservative substances can bring additional dangers for consumers. Therefore, the scientific world began to identify natural substances that on the one hand influence the validity of food, and their sensory characteristics on the other.

The perceived level of risk associated with a food product can influence purchase and consumption decisions.

Nowadays, current trends in food safety are focused on replacing the use of synthetic antioxidants by natural antioxidants, mainly because of the adverse effects of synthetic antioxidants, such as cancer or cardiovascular diseases [2,3].

Additionally, there is still growing concern for natural substances capable of counteracting the effects of oxidative stress that underlie the pathogenesis of numerous diseases, such as neurodegenerative disorders, cancer, diabetes, and atherosclerosis, as well as the negative effects of various harmful factors and drugs [4].

Antioxidants are used to minimise the oxidative changes in food products and as a direct consequence contribute to the extension of the shelf life. Extracts of plant origin can provide natural alternatives that can be used in the food industry instead of synthetic preservation. Antioxidants also provide bioactive properties and bring additional value to the finished products [3,5,6,7]. Additionally, natural and synthetic antioxidants may have negative effects on food quality, causing changes in their nutritional and sensory properties [2,8].

Common foods that are rich in antioxidants include grapes, goji berries, dark-coloured fruits and vegetables, seeds and nuts, green tea, and dark chocolate. In this regard, there have been identified compounds that have both preservative and antioxidant roles, such as benzoic acid, which is found in nature as glucoside, in some fruits (blueberries and plums) and spices (cinnamon and cloves) [9,10,11,12].

Some food products, such as bakery products, dairy products and meat, natural extracts from aromatic plants, spices, and fruit powder, have already been used for antioxidant purposes [2,3,13,14,15,16].

The aim of this paper was to document the current background of using synthetic and natural antioxidants in the food industry with an emphasis on the meat industry for both endogenous and exogenous applications. Additionally, the associated opportunities and risks were identified.

Thousands of scientific papers focusing on antioxidants used in the food industry have been published over the years. Antioxidants used in the food industry can be natural, extracted from different plants, or synthetic. It should be noted that natural antioxidants can be used not only in products of animal origin, but also in those of vegetal origin. In Table 1 it can be seen that antioxidants are found in all foods intended for human consumption, whether they are of animal or non-animal origin. Additionally, most studies have been performed to determine the use of natural antioxidants in the food industry. Another important aspect is that research on antioxidants used in the food industry has been conducted since 1996, but the largest number of works in this field have appeared in the last 3 years.

## 2. Synthetic vs. Natural Antioxidants in the Food Industry

Antioxidants delay or prevent oxidation or neutralise free radicals in food processing to improve flavour, aroma, and colour [17,18]. In this direction, in the food industry, a large number of synthetic or natural substances are used to preserve food products. In the European Union, the use of food additives is regulated by specific laws that describe their purity, chemical properties, the substrate in which they will be applied, the maximum usable doses, the acceptable daily intake, etc. [19,20,21,22,23,24,25,26,27,28,29,30,31,32].

In addition, food preservatives are regulated by Commission Regulation (EC) No 1333/2008 of the European Parliament and of the Council of 16 December 2008 on food additives [33].

There are more and more preservatives used in the food production process in order to prevent their deterioration, which is determined by the action of microorganisms. At the same time, the number of antioxidant substances used in food production has increased, and the final result is the prolongation of product shelf life [2].

Various factors can promote oxidation, including the presence of oxygen, metal ions, moisture, heat, and light; therefore, to prevent or delay oxidation in susceptible foods, oxygen and metal catalysts should be removed and foods must be kept at low temperatures and be protected from light or by adding substances to protect them from oxidative processes [34].

### 2.1. Synthetic Antioxidants in the Food Industry

Given the unstable nature of natural antioxidants, their synthetic homologues are used as preservatives in food products. Additionally, the literature states that synthetic antioxidants are utilised as food preservatives because of their high reactivity and efficiency [17].

Butylated hydroxytoluene (BHT) and butylated hydroxyanisole (BHA) were initially created to prevent oxidative gummification in petroleum. Nonetheless, these chemicals have been employed as antioxidants in foods since 1954, and they are perhaps the most commonly used antioxidants in food products today. Despite their widespread use, the food industry is lobbying for their substitution with natural antioxidants, which are not only more cost effective but also environmentally beneficial. To protect food consumers, their use is regulated by established bodies such as the Food and Drug Administration (FDA) of the United States and the European Food Safety Agency (EFSA), among others. Moreover, it seems that the amount of antioxidants permitted in food is often conditioned by the fat content of the product and is restricted to 0.02% total antioxidants [17]. Comparing the efficiency of natural and synthetic antioxidants is quite challenging, but in general, both work by comparable processes, with their activity depending solely on their polarity and chemical structure [35]. In this direction, synthetic antioxidants were developed to stabilise bulk fats and oil- or lipid-rich foods. On these substrates, they are significantly more effective than α-tocopherol and other naturally occurring antioxidants, which are often less liposoluble. In most circumstances, the maximum legal concentration of synthetic antioxidants is sufficient for food stability, with few exceptions. Besides carotenes, tocopherols and their esters, and sesame seed lignans, natural antioxidants are often more polar than synthetic antioxidants. Hence, natural phenolic antioxidants are typically insufficiently soluble in the lipid phase, limiting their effectiveness in bulk lipids. They are not pure compounds; therefore, the active fraction is often a small proportion of the total addition, whereas synthetic antioxidants are virtually 100 percent pure. Normally, larger quantities of natural antioxidants, such as 0.1–0.5% or even more, are required [35]. In this regard, in a study conducted by Özcan et al. in 2011, it was observed that *Rosmarinus officinalis, Syzygium aromaticum*, and *Cinnamomum zeylanicum* essential oils were less effective compared with BHA when testing the antioxidant effect on hazelnut and poppy oils. On the other hand, *Cinnamomum zeylanicum* essential oil was the most effective in terms of antioxidant efficiency [36].

The main synthetic antioxidants are presented in Table 2, along with the limits imposed by FDA, EFSA, the Joint FAO/WHO Expert Committee, and EC Regulation No. 1333/2008.

### 2.2. Natural Antioxidants: Prospects of Use

Natural extracts of plant origin could be a good alternative to synthetic preservatives, i.e., antioxidants, also adding value to finished products and providing bioactive compounds [3,5,6,7].

Antioxidant functions are associated with lowering DNA damage, malignant transformation, and other parameters of cell damage in vitro as well as epidemiologically with lowered incidence of certain types of cancer and degenerative diseases, such as ischemic heart disease and cataract. They are of importance in the process of aging [37].

Compounds derived from plants are mostly secondary metabolites, out of which a great percentage are phenols or derivatives. These secondary metabolites have various benefits, including antimicrobial properties [38].

Natural pigments, including carotenoids, flavonoids, and anthocyanidins determine the colour of the fruits. These natural pigments are essential secondary metabolites, which play multiple roles in the whole life cycle of plants and are characterised by powerful antioxidant activity [39].

Carotenoids are an unreplaceable class of micronutrients in the human diet and are commonly found in different bacteria, fungi, algae, and plants. More than 800 naturally occurring carotenoids have been found, with yellow, red, orange colour, etc. [40]. The α and β-carotenes belong to the most essential carotenoids in the human body and have a remarkable pharmacological value for health because of their beneficial antioxidant activities [41]. Carotenoid pigments, particularly β-carotene and lycopene, are present in human foods and play a crucial part in keeping people healthy. It is known that β-carotene quenches singlet oxygen and may have powerful antioxidant activity. Thus, it has been reported that β-carotene may decrease the risk of cancer. Epidemiological studies have found inverse relationships between cancer risk and β-carotene ingestion or blood levels [42]. The antioxidant potential of carotenoids can be reduced at high partial pressures of oxygen. In biological systems, carotenoids are unlikely to act as prooxidants; rather, they show a tendency to lose their effectiveness as antioxidants.

In recent years, more and more studies have reported multiple benefits of flavonoids on human health. Flavonoids have gradually become a research hotspot in biology, food science, medicine, and other fields [39]. There are a variety of flavonoids with rich contents in fruits, and more than 5000 different kinds have been identified from plants [43]. Flavonoids have long been a major focus of grape and wine research, as this class of substances not only affects the colour of wine, but also its ageing capacity, astringency, and bitterness [44].

It is already well known that the polyphenols present in plants can play a beneficial role in maintaining the metabolic balance and health of the human body [45,46]. Polyphenols are a very important part of the human diet because they are naturally present in fruits and vegetables. Extracts rich in compounds from edible plants show an antimicrobial capacity against different pathogens.

Furthermore, Mahboubi et al., in 2015, evaluated the antibacterial effects of aromatic plants against food-borne bacteria, including *Staphylococcus aureus*, *Shigella dysenteriae*, *Salmonella typhimuriun, Scherylusis, Epidermia subtilis*, and *Pseudomona aeruginosa* and measured the total amount of phenolic substances in these plants. The results showed that thyme was the most effective against all the bacteria investigated [47].

#### Natural Antioxidant Types Used in the Food Industry

**Figure 1:**
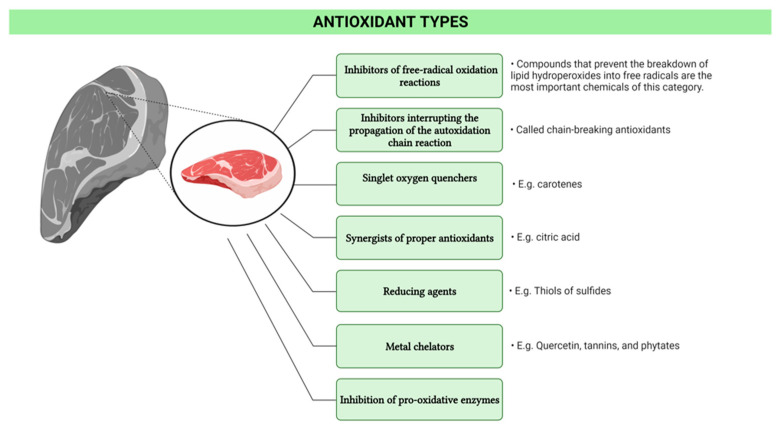
Antioxidant types based on their mechanism of action (illustration made via www.BioRender.com, accessed on 9 March 2023).

Numerous recent investigations have been directed towards the identification of natural antioxidants from various plant sources [2]. In this direction, the main antioxidant types based on their mechanism of action are presented in Figure 1.

Food products can be obtained in conventional, traditional, and organic systems.

In the food industry, various bioactive compounds extracted from plants (exogenous antioxidants) are used, such as phenolic compounds, ascorbic acid, carotenoids, tocopherols, etc., because of their ability to delay food spoilage, improve its sensory qualities, as well as inhibit the growth of some pathogens [48].

Carotenoids are recognised as singlet oxygen quenchers, but can also inactivate peroxyl and hydroxyl radicals. Ascorbic acid is an efficacious radical scavenger and can regenerate the antioxidant form of vitamin E, being a reducer of the tocopheroxyl radical. Phenolic compounds exhibit antiradical activity and promote the activity of antioxidant enzymes. Tocopherol inactivates alkyl and peroxyl radicals, being an effective hydrogen donor, but can also quench singlet oxygen [49].

Some researchers conducted numerous studies intended to compare the use of natural antioxidants from fruit or plant extracts with synthetic antioxidants. For example, the incorporation of fresh mango peel extracts in different food products improved their antioxidant properties, in comparison with butylated hydroxyl anisole (BHA), which is a synthetic one [3,50]. Currently, the food industry is focused in replacing the use of synthetic by natural antioxidants. The present study focused on the use of fennel and chamomile extracts, rich in phenolic compounds, as natural antioxidants in biscuits and compared their performance with a synthetic antioxidant widely used, the butylated hydroxyl anisole (BHA). The complete nutritional profile, free sugars, fatty acids and antioxidant activity were determined immediately after baking and also after 15, 30, 45 and 60days of storage. The results showed that the incorporation of natural and synthetic additives did not cause significant changes in colour or in nutritional value of biscuits when compared with control samples. Both natural and synthetic additives conferred similar antioxidant activity to the biscuits. Therefore, natural additives are a more convenient solution for consumers who prefer foods "free" from synthetic additives. Additionally, natural additives were obtained by aqueous extraction, an environment friendly and safe process [50]. Caleja et al., in 2017, argued that chamomile and fennel extracts are rich in phenolic compounds and can be used as natural antioxidants. Their performance can be compared with a widely used synthetic antioxidant, namely, butylated hydroxyl anisole (BHA) [3]. In other scientific works, it is mentioned that aqueous extracts prepared from chamomile (*Matricaria recutita* L.) and fennel (*Foeniculum vulgare*) can be incorporated as natural antioxidants in different food products [3,14,15]. The content of polyphenols is high in some non-animal by-products (fruits, seeds, peels, etc.), which are available in large quantities and at low costs, but are frequently used only as feed or fertilisers. Their use as food additives could help the industry to solve the environmental problems related to the disposal of these materials and create new sources of natural antioxidants [51]. For example, Nissen et al. used rosemary extract as a natural antioxidant in pork patties in a concentration of 200 ppm. The pork patties were vacuum packaged at 4.5 °C for 10 days and the results showed reduced TBARS and hexanal values [52]. Rosemary extract in a concentration of 300–500 ppm was also used by Sebranek et al. in raw frozen and precooked frozen sausage, with the same results as those mentioned above [53]. Rosemary extract was used to obtain boerewors, which is a South African fresh sausage, in a concentration of 260 mg/kg [54]. For pork patties, Nissen et al. also used coffee extract in a concentration of 50 ppm and green tea extract in a concentration of 200 ppm [52]. Green tea extract was used in a concentration of 300 mg/kg meat for obtaining beef patties. The results obtained after using the natural extract were reduced lipid oxidation and redness loss in raw patties and delayed rancid flavour development in cooked patties [2,55]. Grape seed extract has been successfully used for reducing TBARS values in cooked, frozen, reheated beef patties in a concentration of 0.2 g/kg [56]. Olive leaf extract (200 μg/g meat concentration) was used by Hayes et al. to reduce lipid oxidation in raw and cooked sausages [57]. Efenberger-Szmechtyk et.al. used *Aronia melanocarpa* as a natural preservative in pork products [58].

## 3. Antioxidants Used in the Meat Industry—Current Overview

The use of antioxidants in the meat industry is essential for preserving the quality and extending the shelf life of meat products.

As stated above, studies have shown that natural antioxidants can effectively inhibit lipid oxidation and the formation of off-flavours and odours in meat products. For example, in a study made by Olivas-Méndez et al. the use of natural antioxidants derived from rosemary (*Rosmarinus officinalis*) and garlic (*Allium sativum*) essential oils along with chipotle pepper oleoresin (*Capsicum annum*) in beef hamburgers was evaluated. The researchers found that the addition of the aforementioned extracts improved the microbiological quality and inhibited the lipid oxidation of the beef hamburgers [59]. Similar research was carried out by Mostafa et al. in which the addition of antioxidants derived from green coffee was compared with ascorbic acid in beef meatballs. The result indicated that the green coffee powder could be a potential ingredient for meat product preservation and antioxidant activity at 1000 ppm [60]. Biasi et al. found that goldenberry flour (*Physalis peruviana*) used in Bologna-type mortadella reduced the lipid oxidation and observed an increase in the phenolic compounds after in vitro digestion [61].

Natural antioxidants currently have a significant influence on the meat food system’s level of acceptance and safety, and this trend is expected to continue. Not only do they prevent the food from spoilage due to oxidation, but they also have the potential to effectively inhibit the microorganisms’ growth. In addition, by elevating the naturally occurring levels of antioxidants found in animal products by dietary supplementation, the finished product can be more acceptable to consumers. This subfield of study is experiencing phenomenal growth in published studies [62].

Synthetic antioxidants, on the other hand, are often used in food preservation because of their effectiveness in preventing oxidation. However, as mentioned before, concerns have been raised about the safety of these compounds. The synthetic compounds used for their antioxidant qualities include phenol derivatives (e.g., PG, BHA, BHT), phosphates (e.g., tripolyphosphate), and acids (e.g., HCl, organic). These synthetic antioxidants can be used in conjunction with chemical preservatives (e.g., nitrites, sulfites, benzoic acid, and sorbic acid) [63]. Regarding the current situation, different countries and organisations around the world have established regulations and guidelines for the use of antioxidants in meat products. These regulations vary in terms of the types of antioxidants that are allowed and the maximum levels of use. For example, in the European Union (EU), the use of antioxidants in meat products is regulated by the EU Regulation No. 1831/2008. This regulation sets limits for the use of synthetic antioxidants and lists the authorised synthetic antioxidants that can be added in meat products. In the United States, the use of antioxidants in meat products is regulated by the United States Department of Agriculture (USDA) and the Food and Drug Administration (FDA). The USDA sets guidelines for the use of synthetic antioxidants in meat products, while the FDA regulates the use of natural antioxidants. In Canada, the use of antioxidants in meat products is regulated by the Canadian Food Inspection Agency (CFIA) [64,65,66,67].

### 3.1. Meat and Meat Products Oxidation

**Figure 2:**
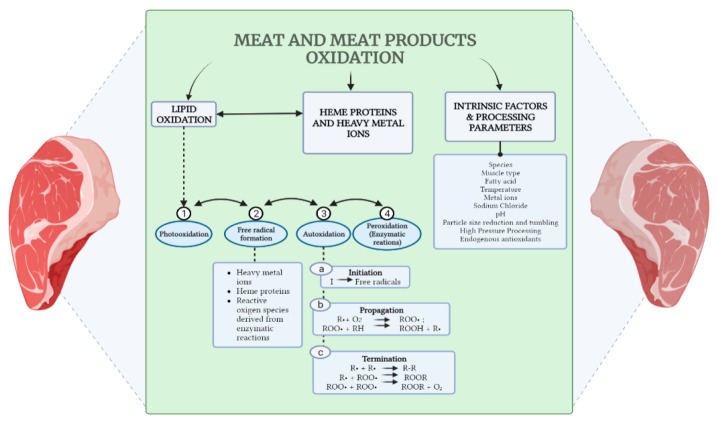
Meat and meat product oxidation in relation to lipid and myoglobin oxidation and other intrinsic and processing factors (illustration made via www.BioRender.com, accessed on 5 February 2023).

Lipids, proteins, pigments, and vitamins in muscle tissue are all biochemical constituents of animal-derived foods that are vulnerable to oxidative reactions that lead to quality loss, such as discolouration, off-flavour development, nutrient loss, textural changes, and the progression of spoilage and/or pathogenicity [68]. Lipids and heme proteins, notably myoglobin, are particularly vulnerable to oxidation (Figure 2). The lipid oxidation process is complex, including several mechanisms that interact with each other. It is generally accepted that the oxidation of lipids in animal-derived foods occurs via an autoxidation route (initiated by free radicals of frequently unknown origin), the photooxidation route, and the enzymatic route (lipoxygenase pathway). The last two routes are often proposed as being involved in the initiation of autoxidation [69,70,71,72,73,74].

Lipid and myoglobin oxidation in meat are linked and both processes are capable of affecting each other (see Figure 2). Metmyoglobin and hydrogen peroxide are produced during the oxidation of oxymyoglobin, which is required to initiate lipid oxidation. Myoglobin’s redox stability is changed by aldehyde lipid oxidation products, leading to increased oxidation of oxymyoglobin and the development of a covalent adduct with myoglobin.

Understanding the responses and mechanisms that may alter the quality and acceptability of meat products and reducing lipid oxidation during handling, processing, and storage is therefore vital.

In order to stop the propagation response during the oxidation process, antioxidants are substances that can provide hydrogen (H·) radicals for combining with other free radicals that are already present. This efficiently reduces rancidity and delays lipid oxidation while preserving the sensory and nutritional qualities of meat products, hence preserving their quality and shelf life [75,76].

Regarding livestock, because of the production of oxygen radicals, an animal’s high metabolic rate makes them more vulnerable to oxidative stress. As explained for sow reproduction, reproductive systems may also be affected by oxidative stress, and gestation may be considered an oxidative stress state because of increased placental mitochondrial activity and ROS generation [77,78,79,80,81,82,83,84,85,86].

Oxidation control in meat systems can take place in either raw or cooked meat systems. In the 1990s, the scientific community paid a great deal of attention to the factors that influence the amounts of endogenous antioxidants in the raw meat system. Prior to this research, the majority of processing-related activities centred on the use of exogenous antioxidants [61].

### 3.2. Natural Antioxidants in Animal Diets

**Figure 3:**
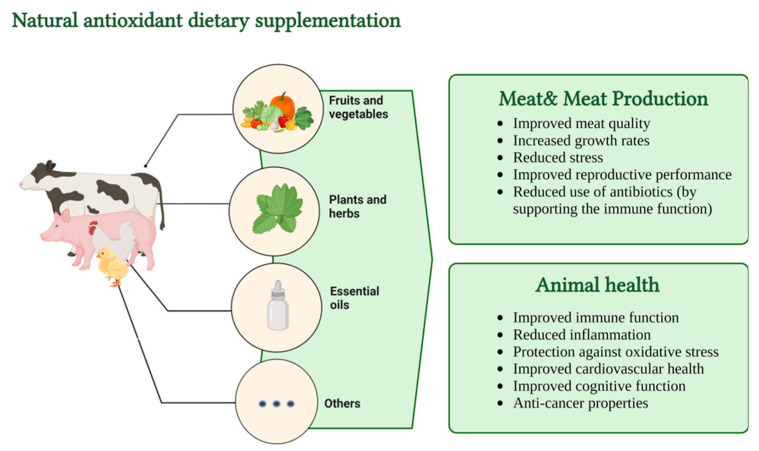
Natural antioxidant dietary supplementation effects (illustration made via www.BioRender.com, accessed on 9 March 2023).

Apart from naturally endogenous antioxidants present in meat, growing evidence suggests that providing animals with diets high in antioxidant molecules can have positive health effects on both the animals and individuals who consume animal products. For example, because of their accessibility and potential for further processing, grape pomace (GP), olive cake, and distillers’ grain wastes have been utilised in animal industries all over the world, but mostly in Europe and countries in south or Central America. Additionally, recent in vivo research investigations on animals have shown that polyphenol-rich by-products derived from the olive oil and wine industries enhanced the antioxidant capacity, meat quality, and welfare of livestock including swine, poultry, and lambs. This trend is associated with the fact that these by-products contain antioxidants and other bioactive compounds such as essential fatty acids, vitamins, and minerals. At the same time, the costs are lower compared with cereal grains or protein meals and the current by-product-processing technologies are based on the prevention of animal diseases caused by microbial development (Figure 3). Another very important advantage is related to the current problems associated with ecology, limiting the discharge of by-products in terrestrial and aquatic ecosystems [77,87,88,89].

Although there are multiple advantages, antioxidants can increase the level of heavy metals and chemical residues in the meat. In addition, they are an opportune environment for the development of moulds and fungi, a fact that can have consequences to the productivity and health of the animals [77].

#### 3.2.1. Swine

Endogenous antioxidants are naturally present in pork meat (as in other meat types) and include enzymes such as catalase, superoxide dismutase, and glutathione peroxidase (GPx), and non-enzymatic antioxidants such as vitamin E (α-tocopherol), vitamin C (ascorbic acid), and carotenoids [88].

Several studies have proven that the quality and shelf life of meat can be enhanced by supplementing animal diets with natural antioxidants prior to slaughter. Delaying lipid oxidation, colour loss, and microbial development are thus among the favourable impacts of natural antioxidants on meat quality [90,91,92].

In this regard, the diet of monogastric animals has a greater effect on the stability of meat, as indicated by some researchers. For example, Guo et al. concluded that the supplementation of feed with 200 UI of alpha-tocopherol acetate (VE)/kg for 6 weeks before slaughter improves lipid stability and pork quality. In another study, Onibi et al. used two levels of α-tocopherol acetate (200 mg/kg^−1^ and 500 mg/kg^−1^) and two basal diets in pigs for 35 days. The authors concluded that there was an increase in the muscle tocopherol levels depending on the diet fed [38,93,94].

The quality of meat products can also be enhanced by plant polyphenols, which reduce the negative effects of lipid peroxidation by lowering malondialdehyde concentrations and raising tocopherol levels in tissues. It has been suggested that several phenolic substances, including genistein, daidzein, soybean isoflavone, and ferulic acid, have an impact on animal metabolism. Moreover, they improve nutrient absorption and availability by increasing digestive secretion, such as saliva and enzymes. It is also important to mention that through their feed, animals in intensive breeding systems are regularly subjected to the oxidation of fatty acids (peroxidation). In this direction, Rossi et al. investigated the supplementation of the pig diets with plant extracts from *Lippia* spp. on the carcass parameters, meat quality, collagen properties, and oxidative stability of *Longissimus dorsi* (LD) muscle. Pigs given the plant extract had lower levels of lipid oxidation in their raw LD than controls [95].

Another effective natural source of antioxidants is green tea extract, which is rich in catechins, a type of polyphenol. The supplementation of green tea extract in the diet of pigs has been shown to increase the level of antioxidants in pork meat, such as vitamin E and glutathione (GSH), and decrease the level of lipid oxidation markers. A study performed by Norkeaw et al. examined the influence of green tea extract supplementation on the growth performance, meat quality, and oxidative stability of the *Longissimus thoracis et lumborum* (LTL) muscle in growing-finishing pigs. The experimental diets for each period consisted of a standard diet (control) and a standard diet supplemented with 500 mg/kg of green tea extract. The addition of green tea extract to the diet had no effect on animal performance or meat quality criteria. However, green tea extract supplementation increased the oxidative stability of LTL muscle throughout 6 days of storage at 4 °C. Moreover, the addition of green tea extract had no effect on the sensory attributes [96].

On the other hand, there were also studies in which no effect on the lipid oxidation was found after administering an antioxidant-supplemented diet in pigs. For example, O’Grady et al. fed pigs with three grape extract treatments (100, 300, and 700 mg/kg feed) and bearberry (100, 300, and 700 mg/kg) for 56 days pre-slaughter in order to observe the oxidative stability and quality of raw meat. The study concluded that none of the treatments significantly reduced lipid oxidation in tissue homogenates [97].

Other natural antioxidant sources used in swine supplemented diet formulations are shown in Table 3.

#### 3.2.2. Ruminants

Vitamin E, C, B complex, and beta-carotene are the most studied dietary antioxidants. Fruits and vegetables provide vitamin C and carotenoids, whereas whole grains and high-quality green vegetables contain vitamin E. Farm animals acquire vitamin E from pasture, legumes, forage crops, silage, yeasts, and bioactive plants and herbs. As a powerful lipid-soluble antioxidant, vitamin E has chain breaking properties within the cell membrane and prevents membrane fatty acid peroxidation. It is also important to mention that natural tocopherol (D-α-tocopherol) is more effective at transitioning from feed to muscle compared with synthetic tocopheryl acetate, which is a mix of eight stereoisomeric forms of vitamin E. Both can be supplemented to meat and milk-producing animals. In some studies, regarding the addition of vitamin E to the livestock diet, the effect was notable in beef, where delayed discolouration and lipid oxidation are the principal effects. In addition, it was concluded that a threshold level of α-tocopherol in muscle ensures a noticeable effect and that the dietary methods for achieving this must take into account the tocopherol status of calves entering the feed yard as well as the length and amount of supplementing [62,76,101].

Liu et al. studied the lipid oxidation in samples of cooked *Gluteus medius* from Holstein steers fed four doses of α-tocopheryl acetate (0, 250, 500, and 2000 mg/steer daily) for 42 or 126 days. The α-tocopherol concentrations rose in both raw and cooked muscle and it was concluded that the ingestion of 500 mg of α-tocopheryl acetate per day for 126 days results in 3.4 g of α-tocopherol per gram of cooked *Gluteus medius*. Similar results were obtained by Galvin et al. in a study on the effect of dietary vitamin E supplementation on the oxidation of cholesterol in vacuum-packaged, cooked, refrigerated, and frozen beefsteaks. It was observed that vitamin E increased the oxidative stability of the meat, as evidenced by a significant decrease in TBARS following supplementation [102,103].

Faustman et al. investigated the pigment and lipid oxidation in freshly ground sirloin from unsupplemented and vitamin E-supplemented (370 I.U./head/day) Holstein steers. They concluded that pigments and lipids oxidised the least in meat containing greater than approximately 0.3 mg α-tocopherol per 100 g of tissue [104].

In another study, Mercier et al. investigated the effect of finishing diets on lipid and protein oxidation in beef homogenates. Pasture-finished animals had a dramatically reduced meat lipid oxidation. The same feeding system increased SOD but decreased GPx activity. Additionally, it was observed that pasture-fed beef had more vitamin E [105].

Regarding other ruminant species, Vlahova-Vangelova et al. investigated how Siberian larch dihydroquercetin and dry-distilled rose petal (DDRP) supplementation of lambs’ diets influenced Bulgarian dairy sheep meat quality. DDRP supplementation boosted muscle sensory parameters, carotenoids, and essential amino acids but not tocopherols. Moreover, DDRP had higher sterol levels in muscle and adipose tissues but not in the liver tissue [106].

It is also important to mention that research suggests that grass-based diets can increase beef’s fatty acid (conjugated linoleic acid (C18:2) isomers, trans vaccenic acid (C18:1 t11), a precursor to conjugated linoleic acid, and omega-3 (n-3)) composition and antioxidant content, while palatability may vary. Compared with grain-fed, grass-fed diets raise the level of vitamin A and E precursors and cancer-fighting antioxidants including GSH and SOD [107].

Other natural antioxidant sources used in ruminants’ supplemented diet formulations are shown in Table 4.

#### 3.2.3. Poultry

Despite having a low lipid content, poultry meat is particularly susceptible to oxidative deterioration due to its comparatively high concentration of PUFA. Broiler meat’s vulnerability to lipoperoxidation rises as the PUFA content of the feed and the PUFA/saturated fatty acid balance in the carcass increases [113,114].

According to research, broiler diets could be supplemented with vitamin E and oregano essential oil as natural antioxidants to prevent the oxidation of unsaturated fatty acids in muscle cell membranes. Additionally, broilers should ingest more vitamin E than what is necessary for their nutritional requirements. From this perspective, the necessity to investigate substitute compounds with antioxidant activity to preserve meat and meat products is highlighted by the fact that as the amount of vitamin E in the diet increases, so do production costs [115,116].

In a study performed by Avila-Ramos et al., two doses of vitamin E (10 or 100 mg/kg^1^ of feed) or oregano essential oil (100 mg/kg^1^ of feed) was administered to broilers. Additionally, honey (3%) or BHT 0.02% was applied to the control treatment group (10 mg/kg^1^ vitamin E). In comparison to the other antioxidant treatments, the 10 mg/kg^1^ vitamin E treatment resulted in greater malondialdehyde levels in meat for all storage days. On the other hand, vitamin E supplementation at the same concentration as oregano oil, 100 mg/kg^1^, resulted in a greater antioxidant impact on the meat [115]. In another study, five dosages of vitamin E (30, 90, 150, 210, and 270 mg/kg feed) were added to the diets of broilers (42–54 days of age). The meat brightness increased linearly along with the vitamin doses, whereas yellowing, cooking loss, and lipid peroxidation were unaffected by treatments. However, some drawbacks regarding the use of vitamin E are mentioned in the literature, for example, the low bioefficiency and its synthetic origins in the context of a growing interest on more natural alternatives. In this regard, the use of polyphenols was also researched. For example, Sáyago-Ayerdi et al. tested the efficiency of dietary GP concentrate and concluded that it significantly inhibited the lipid oxidation of raw and cooked breast chicken patties [116,117]. Aditya et al. also obtained promising results on the growth performance, blood profile, meat quality, and apparent total tract digestibility of nutrients by using GP. The study concluded that the supplementation had quadratic effects on body weight gain throughout early growth stages and was helpful in reducing blood cholesterol level and increasing meat quality indices in broilers [118]. Other natural antioxidant sources used in poultry supplemented diet formulations are shown in Table 5.

### 3.3. Natural Antioxidants Used in Meat and Meat Products

Thermal, non-thermal, and various conservation methods such as irradiation, high hydrostatic pressures, and salt incorporation promote the oxidation of lipids and proteins via Strecker reactions, the release of metals such as iron, the exposure of fatty acids by cell membrane disruption, and the increase in the redox potential of products. Antioxidants, on the other hand, exert their effects through the resonance of their conjugated double bonds and the ease with which they transfer electrons, create bonds, or donate hydrogen [122].

**Figure 4:**
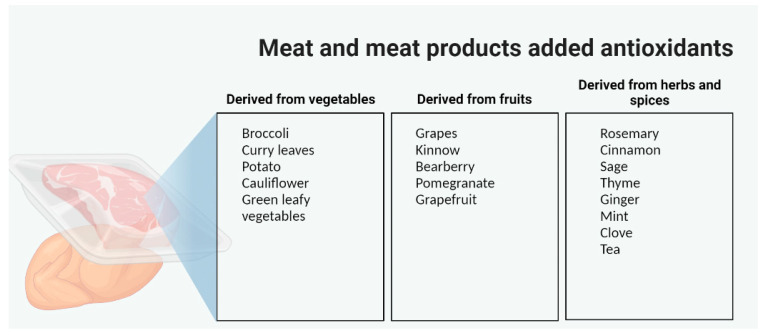
The main categories of natural antioxidants tested in meat and meat products (illustration made via www.BioRender.com, accessed on 9 March 2023).

Traditional methods for controlling lipid oxidation in meat and meat products have involved the addition of antioxidants during processing. Some of the additional components, such as ascorbic acid and carnosine, are naturally present in meat, while others are synthetic or sourced from plants [61]. Various types of plant-derived antioxidants have been extracted from fruits, herbs, and spices to reduce lipid oxidation (Figure 4) [81].

Phenolic compounds, carotenoids, and essential oils are the primary classes of natural phytochemicals able to exert antioxidant action. Phenolic compounds are a varied group of secondary metabolites that are extensively dispersed in plant-based foods. Because of their capacity to absorb UV radiation and deactivate and stabilise free radicals by integrating them into their aromatic rings, phenolic compounds are regarded as potent antioxidants [123,124].

Moreover, Sebranek et al. investigated the use of synthetic antioxidants such as butylated hydroxy anisole (BHA) and butylated hydroxyl toluene (BHT) in comparison with rosemary extract in pork sausages. The researchers found that the rosemary extract was more effective than BHA and BHT in preventing the increase of thiobarbituric acid-reactive substance (TBARS) values [53]. Similar results were also obtained by Patiero et al. (2014), concluding that tea and grape extracts could replace BHT in terms of efficiency [125].

On the other hand, Yu-Yue Qin et al. conducted a similar study in 2013 to test the antioxidant potential of pomegranate rind powder extract, pomegranate juice, and pomegranate seed powder extract in comparison with BHT. The researchers concluded that BHT showed the highest efficiency, followed by pomegranate rind powder extract, pomegranate juice, and pomegranate seed powder extract [126].

In this regard, some natural antioxidant sources used in meat products are shown in Table 6.

Considering the aforementioned, from the point of view of legislative regulations, the European Commission has approved rosemary extract (E392) as a food additive for meat products, up to 150 mg/kg (EU 1333/2008 and 1129/2011), along with several other phenolic compounds as flavourings (EU 872/2012). Globally, rosemary extract is also permitted in China, Japan, Australia, New Zealand, and the US. In addition, considering the contact with food, according to Regulation (EU) No 450/2009, it is necessary to include phenolic compounds in the list of permitted additives, which limits their widespread use [124].

Another problem associated with the addition of phenolic compounds in meat products is regarded to their interaction with lipids, proteins, and carbohydrates, as well as affecting pH or sensory characteristics. On the other hand, although phenolic compounds are considered to limit the digestibility of proteins and amino acids, these effects have not been observed in meat [124].

Microorganisms and plants (including acerola, cabbage, carrots, and paprika) synthesise lipophilic, coloured carotenoids. Carotenoids have a polyisoprenoid structure and near-bilateral symmetry around the central double bond. Carotenoids create resonance-stabilised radical adducts with peroxyl radicals. They can prevent cell wall lipid degradation by scavenging peroxyl radicals. Lipophilic long unsaturated alkyls make them easy to integrate into fatty products. Unsaturated double bonds boost carotenoids’ antioxidant activity. The β-carotene and lycopene, which have eleven conjugated double bonds, quench singlet oxygen better than lutein, with ten conjugated double bonds [139].

Carotenoids are a common type of natural pigments used as food colourants (red to yellow). Their antioxidant activity and non-toxicity make them good natural food additives, with the only associated problem being that they can alter the appearance of fresh raw meat or mask the signs of spoilage [139,140].

Essential oils are aromatic, volatile oily liquids extracted from different plant components. It is well known that the antioxidative activity of a certain essential oil can be related to the interaction between its major and minor constituents and has been consistently related to the presence of phenols, such as carvacrol, eugenol, and thymol, which serve as free radical scavengers and hydrogen donors.

The lack of legislative framework, the wide diversity of meat-based products and targeted pathogens, the limited number of case-specific application procedures, and the doubtful universal efficacy of those that have been applied continue to be significant impediments [124].

## 4. Future Trends and Associated Risks of Natural Antioxidant Application in Meat and Meat Products 

Because of the link between food and chronic diseases, customers prefer healthy foods such as plants and vegetables. In this respect, most current research on redox reactions in meat systems has been focused on discovering plant-based antioxidant molecules and assessing their protective effects. Problems associated with this phenomenon are caused by variations in application methods, antioxidants used, meat systems, and, possibly most importantly, the lack of in-depth research. This can lead to uncertainty, variable results, and major safety issues resulting from not knowing the nature, concentration, and molecular basis of bioactive substances. As a result, the possibility of using some natural antioxidants on a large scale is reduced [141].

Another debatable topic is represented by the tendency to use natural antioxidants against synthetic ones. In certain situations, not all natural sources of antioxidants have the expected effect from the quality and safety point of view. These aspects are especially related to dose, composition, and origin of the natural antioxidant source. Comparatively, synthetic antioxidants approved by law and used in the recommended doses do not represent a health risk. On the other hand, it should be mentioned that the use of synthetic antioxidants is usually limited in certain categories of meat products by the legislative bodies. At the same time, in some natural sources of antioxidants, it is also possible to identify compounds that need to be replaced from conventional meat products. For example, green-leafed plants contain a high level of nitrites and nitrates [141,142,143,144,145].

Another related problem is the polarity of the antioxidants and the fact that natural antioxidants show a wide variability. From this point of view, it can be difficult to replicate the results [141].

From the trends point of view, the use of natural antioxidants can be the basis for functional formulations or novel foods. On the other hand, the association of meat consumption with the occurrence of some diseases can decrease the general consumption, regardless of the additive types [141].

Furthermore, meats and meat products differ in oxidative responses, intensity, and effects. In this context, as some authors suggested, it seems vital to carry out an initial oxidation risk assessment and to determine if the time and effort required for creating and deploying an antioxidant strategy are needed. If they prove to be necessary, the safety, availability, efficiency, sustainability, and costs must be evaluated for natural antioxidant selection. Afterwards, the method of application can be decided—endogenous or exogenous [141,146].

In addition, lipid oxidation also has positive effects on meat and meat products, contributing to the formation during ripening or dry-cured stages of some derived compounds with the typical aroma of meat products appreciated by consumers [68]. Thus, it is difficult to find an antioxidant or a mixture of antioxidants that mitigates the undesirable effects of lipid oxidation on the one hand and, on the other hand, favours the formation of compounds with a pleasant aroma.

## 5. Conclusions

The natural antioxidants’ efficacy was demonstrated in numerous in vitro and in situ studies. Besides their effects against oxidation and spoilage, there are also studies on their antibacterial potential. Furthermore, the use of some natural sources of antioxidants can protect the environment, also being associated with low costs. In this regard, numerous wastes obtained by different industries (e.g., GP, distillers’ grain waste) are proved to be efficient in a variety of food substrates.

Despite these positive aspects, there are some associated challenges that limit their widespread use in the food industry. For example, supplementing diets with antioxidants can increase the level of heavy metals or chemical residues in meat. Moreover, some natural sources of antioxidants may be serve as appropriate substrates for the growth of moulds and fungi, which can have consequences for animal health.

On the other hand, for meat and meat products, there is a wide variability in the intrinsic desirable characteristics which are sometimes modulated by processes such as lipid oxidation and the associated flavour development. Thus, identifying a natural antioxidant that mitigates the negative effects of lipid oxidation but still preserves the flavour development can be difficult. Additionally, the variability in research methods, meat systems, and targeted antioxidants (including types, dose, origin, polarity, etc.) makes it impossible to draw a relevant conclusion on the overall efficacy. It is also important to mention that regardless of the food product origin, a preliminary evaluation should be carried out before applying a natural antioxidant, as this type of treatment is not always required.

On the other hand, new trends are converging towards the formulation of functional foods, natural antioxidants being a viable opportunity in this direction.

In conclusion, the prospects for the use of natural antioxidants are promising only when overcoming the challenges mentioned above.

## Figures and Tables

**Table 1:** Bibliometric analysis according to the “Web of Science” database.

Field of Research	Number of Scientific Publications
Natural antioxidants used in food industry	3813
Natural antioxidants in animal origin food	74
Natural antioxidants in non-animal origin food	8
Natural antioxidants in meat	2098
Natural antioxidants in meat products	1324
Natural antioxidants in fishery products	28
Natural antioxidants in milk	826
Natural antioxidants in dairy products	264
Natural antioxidants used to obtain plant-based foods	22

**Table 2:** The limits imposed by the regulatory bodies for the main synthetic antioxidants used in the food industry [14,33].

Synthetic Antioxidant	Limits Imposed by the Main Regulatory Bodies		Food Matrices
BHA	FDA	-	Powder milk, fats and oils, potato, chewing gum, cereals, bakery products, meat products, pastries, spices, mustard, biscuits, cakes, etc.
	EFSA	-	
	Joint FAO/WHO Expert Committee	200 mg/kg	
	EC Regulation No. 1333/2008	25–400 mg/kg	
BHT	FDA	-	Fats and oils, pastries, chewing gum, meat products, potato, spices, milk products, etc.
	EFSA	400 mg/kg	
	Joint FAO/WHO Expert Committee	100 mg/kg	
	EC Regulation No. 1333/2008	100–400 mg/kg	
Propyl Gallate (PG)	FDA	-	Vegetable oil, meat products, potato, chicken soup base, spices, chewing gum, milk products, etc.
	EFSA	-	
	Joint FAO/WHO Expert Committee	200 mg/kg	
Octyl Gallate (OG)	FDA	-	Oils and fats, snack foods, dairy products, cereals, meat products, etc.
	EFSA	-	
	Joint FAO/WHO Expert Committee	200 mg/kg	
Dodecyl Gallate (DG)	FDA	-	Oils and fats, snack foods, dairy products, cereals, meat products, etc.
	EFSA	-	
	Joint FAO/WHO Expert Committee	200 mg/kg	
EDTA	FDA	75 ppm	Oils and fats, margarine, mayonnaise, canned shellfish, processed fruits and vegetables, salad dressing, etc.
	EFSA	-	
	Joint FAO/WHO Expert Committee	-	
	EC Regulation No. 1333/2008	75–250 mg/kg	
TBHQ (tertiary butylhydroquinone)	FDA	-	Milk and milk products, oils and fats, cereals, meat and meat products, spices, chewing gum, bakery products, potato, fish and fish products, seafood, soups, etc.
	EFSA	-	
	Joint FAO/WHO Expert Committee	120 mg/kg	
	EC Regulation No. 1333/2008	25–400 mg/kg	

**Table 3:** Literature review on the effects of some natural antioxidant sources by diet supplementation in swine.

Origin	Main Antioxidants	Species/Category	Dose/Treatment	Tested Effect	Results	Ref.
Avocado	Vitamin C, vitamin E, and the carotenoid lutein	Swine	Control (fed basic diet) and treated pigs (fed on avocado-supplemented diet)	Oxidative stability of lipids and proteins during pork processing	Cooked and chilled loins from treated pigs had much lower concentrations of lipid and protein carbonyls than control counterparts. Dietary avocado was unable to prevent the oxidation of tryptophan and thiols.	[98]
GP	Polyphenols (Gallic acid, its 3- and 4-β-glucopyranosides, trans-caftaric acid, cis- and trans-coutaric acids, 2-hydroxy-5-(2-hydroxyethyl)phenyl-β-glucopyranoside, catechin, epicatechin and procyanidin B1), resveratrol and anthocyanins	Swine (weaned piglets)	Standard or experimental diet for 30 days	Potential beneficial effects on welfare, productivity and meat quality	Reduced levels of TBARS and protein carbonyls in the GP group compared to the control group in piglets given the experimental diet (lower oxidative stress-induced damage to lipids and proteins)	[88]
		Fattening-finishing pigs	Control and 5% for 24 days	Analyse the presence of polyphenols from GP in IPEC cells, in the duodenum and colon of piglets fed diets with or without 5% GP, and compare and correlate their in vitro and in vivo absorption.	5% GP increased the total antioxidant status (TAS) and decreased lipid peroxidation (TBARS) in both duodenum and colon, and increased SOD activity in duodenum and CAT and GPx activity in colon	[99]
*Lippia* spp. plant extract (PE)	Rosmarinic acid, luteolin, quercetin, kaempferol, limonene, carvone	Swine	Control and experimental treatments with 5 mg/kg feed from weaning to slaughter (166 days)	Carcass characteristics, meat quality, collagen characteristics, oxidative stability, and sensory attributes of LD	Raw LD of pig fed PE showed lower (*p* < 0.001) lipid oxidation levels than controls	[95]
Green tea extract	Epigallocatechin gallate, quercetin, kaempferol, gallic acid	Swine (growing-finishing pigs)	Control and standard diet supplemented with green tea extract at 500 mg/kg diet	Growth performance, meat quality and oxidative stability of LTL	Green tea extract improved the oxidative stability of LTL muscle during storage at 4 °C for 6 days	[96]
Oregano essential oil (OEO), quercetin or vitamin E	OEO—carvacrol, thymol, rosmarinic acid, and other phenolic compounds	Swine (Large White×Landrace)	Control or basal diet with 200 mg vit. E/kg (positive control), 25 mg OEO/kg or 25 mg quercetin/kg for 4 weeks	Live body weight loss, carcass characteristics, meat quality and antioxidant status of pigs after transportation	The OEO and quercetin groups exhibited lower levels of TBARS and ROS in serum, muscle, and liver than the control group (*p* < 0.05), while the vitamin E group only had lower levels in serum	[100]

**Table 4:** Literature review on the effects of some natural antioxidant sources by diet supplementation in ruminants.

Origin	Main Antioxidants	Species/ Category	Dose/Treatment	Tested Effect	Results	Ref.
Whole dried citrus pulp	Naringin, hesperidin, lutein, zeaxanthin, ferulic acid	Ovine (lambs)	Barley-based concentrate diet, or a concentrate-based diet including 35% whole dried citrus pulp to partially replace barley	Antioxidant status of lamb tissues	The muscle from lambs fed whole dried citrus pulp displayed lower TBARS values, which negatively correlated with the concentration of α-tocopherol in muscle	[108]
GP	Polyphenols (Galic acid, its 3- and 4-β-glucopyranosides, trans-caftaric acid, cis- and trans-coutaric acids, 2-hydroxy-5-(2-hydroxyeth-yl)phenyl-β-glucopyranoside, catechin, epicatechin and procyanidin B1), resveratrol and anthocyanins	Ovine (lactating ewes and lambs)	4 treatments—linseed oil (Control) supplemented with Vitamin E or two levels of GP	Testing the effect in lactating ewe rations on meat quality and fat composition of their suckling lambs	GP improved the water holding capacity of the meat. The use of GP as a dietary supplement in ewe rations would not have negative effects on meat from suckling lambs	[109]
Rosemary distillation residues	Carnosic acid, carnosol, rosmarinic acid, and other phenolic compounds	Ovine (lambs)	Control group—600 g of hay, which was substituted by 600 g of pellets containing 60 and 87% of rosemary distillation residues; all animals received 600 g of concentrate	Lamb meat quality, oxidative stability and fatty acid profile	Rosemary distillation residues increased vitamin E, improving lamb meat’s fatty acid profile and oxidative status	[110]
Plant extracts rich in polyphenols—rosemary (*Rosemarinus officinalis*), grape (*Vinis vitifera*), citrus (*Citrus paradisi*) and marigold (*Calendula officinalis*) and vitamin E	Rosemary—Carnosic acid, carnosol, rosmarinic acid, and other phenolic compounds Grape—Polyphenols (Gallic acid, its 3- and 4-β-glucopyranosides, trans-caftaric acid, cis- and trans-coutaric acids, 2-hydroxy-5-(2-hydroxy-ethyl) phenyl-β-glucopyranoside, catechin, epicatechin and procyanidin B1), resveratrol and anthocyanins Citrus—naringin, hesperidin, vitamin C, limonoids Mariogold—quercetin, kaempferol, lutein, zeaxanthin, chlorogenic acid	Bovine (culled cows)	Polyunsaturated fatty acids (PUFA) -rich cull cow diets with vitamin E (2.8 g/animal/day) or vitamin E plus plant extracts rich in polyphenols	Oxidative stability of longissimus thoracis and semitendinosus steaks	Vitamin E plus the tested extracts proved to be more effective than vitamin E alone for the most deleterious beef packaging.	[111]
Palm oil or canola oil	Palm oil—tocotrienols, carotenoids (beta-carotene), and phenolic compounds (catechins, quercetin) Canola oil—vitamin E (tocopherols and tocotrienols), phytosterols, and polyphenols	Goats (Kacang kid)	Diets with 65% concentrates (including either 3% canola or 3% palm oil) and 35% roughage	Performance, plasma, and tissue fatty acid profile	Palm oil-fed kids had greater liver and LL lipid oxidative substances than canola-fed kids	[112]

**Table 5:** Literature review on the effects of some natural antioxidant sources by diet supplementation in poultry.

Origin	Main Antioxidants	Species/ Category	Dose/Treatment	Tested Effect	Results	Ref.
Olive oil industry waste—semi-solid olive cake (pOC)	Phenolic compounds (such as oleuropein and hydroxytyrosol), tocopherols, and carotenoids	Poultry (Ross 308)	Control, diet supplemented with a low dose of pOC (82.5 g/Kg, L-pOC), diet supplemented with a high dose of pOC (165.0 g/Kg, H-pOC)	Quality characteristics, antioxidant capacity, oxidative status, and consumer acceptability of chicken meat	The greatest pOC level improved meat’s antioxidant status and oxidative stability.	[86]
Extra-virgin olive oil	Hydroxytyrosol, tyrosol, and oleocanthal, vitamin E (tocopherols and tocotrienols)	Poultry (Hubbard strain)	Diet containing sunflower oil; diet containing lard, and diet containing extra-virgin olive oil	The antioxidant effect	Extra-virgin olive oil supplementation significantly lowered lipid peroxidation by boosting antioxidant defence system	[119]
Olive mill wastewater phenolic concentrate	Hydroxytyrosol, tyrosol, oleuropein, verbascoside, ligstroside	Poultry (Ross 308)	Control diet, control diet supplemented with 4.8% olive mill wastewater extract and control diet supplemented with 9.9%	Lipid and protein oxidation and oxidative stability during storage	Olive mill wastewater extract delayed lipid and protein oxidation and increased antioxidant activity during storage	[120]
GP concentrate	Polyphenols (Galic acid, its 3- and 4-β-glucopyranosides, trans-caftaric acid, cis- and trans-coutaric acids, 2-hydroxy-5-(2-hydroxyeth-yl)phenyl-β-glucopyranoside, catechin, epicatechin and procyanidin B1), resveratrol and anthocyanins	Poultry	0, 30 and 60 mg/kg	Lipid peroxidation levels (TBARS) and antioxidant capacity (ABTS method) of raw and cooked chicken breast meat	Dietary GP concentrate effectively inhibited lipid oxidation of raw and cooked breast chicken patties compared to samples from chickens fed the control diet at 20 days and long-term frozen storage (6 months)	[116]
Thyme (TEO) and rosemary (REO) essential oils	TEO—thymol, carvacrol, linalool, camphene, caryophyllene. REO—carnosic acid, carnosol, rosmarinic acid, ursolic acid, camphor.	Poultry (Ross 308)	0, 150 mg kg^−1^ TEO, 300 mg kg^−1^ TEO, 100 mg kg^−1^ REO and 200 mg kg^−1^ REO	Lipid oxidation, water activity, pH, colour and microbial quality	REO and TEO extracts significantly decreased TBARS levels.	[121]

**Table 6:** Literature review on the effects of some natural antioxidant sources in meat products.

Type	Product	Origin	Main Antioxidants	Dose/ Treatment	Tested Effect	Result	Ref.
PORK	Sausages	Banana inflorescences (male flowers extracts)	Catechins, epicatechins, proanthocyanidins (procyanidin B2), carotenoids (α-carotene, β-carotene, lutein), flavonoids (kampferol, quercetin, rutin), vitamin C, vitamin E	0, 0.5, 1, 1.5, and 2% EMF	Physicochemical, oxidative, and sensory characteristics assessment	The treatments had strong antioxidant action without affecting pH, aw, or colour. Additionally, extracts up to 2% did not impair product sensory quality.	[127]
	Raw ground meat	Pomegranate rind powder extract (PRP), pomegranate juice (PJ), and pomegranate seed powder extract (PSP)	Punicalagins Ellagic acid Anthocyanins Vitamin C Vitamin E	Control group, 20% PRP, 20% PJ, 20% PSP, and 20% BHT	pH, microbiological, TBARS value, peroxide value, colour, sensory evaluation	BHA was the most effective, followed by PRP	[126]
	Liver pâté	Green tea, chestnut, and grape extract	Green tea - Epigallocatechin gallate (EGCG) Catechins Polyphenols Chestnut - Tannins Ellagic acid Gallic acid Vitamin C Grape extract—Resveratrol Quercetin Anthocyanins Catechins Vitamin C	Control, BHT (200 mg/kg), tea extract (1000 mg/kg), chestnut extract (1000 mg/kg) and grape seed extract (1000 mg/kg)	Physical analysis, lipid oxidation (peroxide index and TBARS), fatty acids methyl esters, volatile compounds	All the natural sources of antioxidants were effective. The lower TBARS values were obtained in green tea and grape extracts	[125]
	Ham	Plum juice concentrate, plum powder	Plum juice concentrate—Anthocyanins Polyphenols Vitamin C Vitamin E Plum powder—Anthocyanins Polyphenols Chlorogenic acid Quercetin Vitamin C	(20% *w*/*w*) fresh plum juice concentrate, dried plum juice concentrate, or spray dried plum powder at 2.5% or 5% and control	Cook loss, vacuum-package purge, Allo–Kramer shear force, TBARS, proximate analysis, objective colour, sensory panel colour and sensory attributes	No differences in lipid oxidation among treatments	[128]
	Burgers	Red grape pomace extracts (Methanolic extraction and High-Low Instantaneous Pressure)	Polyphenols Resveratrol Anthocyanins Proanthocyanidins Vitamin C	0.06 g/100 g final product of the extract	pH, microbial spoilage, lipid oxidation and colour	High-Low Instantaneous Pressure was more effective on lipid oxidation and could be a possible alternative to optimise the grape extracts for preservative purposes	[129]
BEEF	Patties	Seasonings derived from wine pomace	Anthocyanins (e.g., malvidin, delphinidin), flavonols (e.g., quercetin, kaempferol), stilbenes (e.g., resveratrol), carotenoids (β-carotene), vitamin C	Three different seasonings obtained from wine pomace, 2 g/100 g compared with sulphites in different storage conditions	Lipid oxidation in raw and cooked beef patties	The seedless wine pomace seasoning inhibited lipid oxidation best under the three circumstances evaluated.	[130]
		Grape seed extract, oleoresin rosemary, water-soluble oregano extract	Grape seed extract: Proanthocyanidins, Resveratrol, Flavonoids, Vitamin E, Linoleic acid. Oleo-resin rosemary: Carnosic acid, Carnosol, Rosmarinic acid. Water-soluble oregano extract: Rosmarinic acid, Thymol, Carvacrol, Flavonoids.	Grape seed extract (GS; 0.01 and 0.02%), oleoresin rosemary (0.02%) and water-soluble oregano extract (0.02%)	Antioxidant, sensorial and physico-chemical efficiency	Grape seed extract provided a modest level of defence against oxidation	[131]
	Meatballs	Pomegranate peel nanoparticles	Ellagic acid, punicalagins, flavonoids (e.g., quercetin), β-carotene, vitamin C	1 and 1.5% and compared with 0.01% BHT and control	Antioxidant and antimicrobial efficiency	The treatments improved meatball cooking, microbiological quality, and lipid oxidation.	[132]
	Sausages	Grape seed extract	Proanthocyanidins Resveratrol Flavonoids Vitamin E Linoleic acid	Grapeseed extract (100, 300, and 500 ppm), ascorbic acid (AA, 100 ppm of fat) and propyl gallate (PG, 100 ppm of fat)	Antioxidant, sensorial and physico-chemical efficiency	TBARS values did not change significantly for the grape seed extract over the tested period	[133]
	Roast beef	Plum juice concentrate, plum powder	Polyphenols Vitamin C Beta-carotene Chlorogenic acid	2.5 or 5% fresh plum juice concentrate, 2.5 or 5% dried plum juice concentrate, or 2.5 or 5% spray dried plum powder and control	Vacuum-packaged purge, Allo-Kramer shear force, lipid oxidation (TBARS), colour space values, and sensory attributes	The tested extracts were effective on lipid oxidation	[128]
POULTRY	Chicken meat patties	Pomegranate peel and bagasse powder and their extracts	Pomegranate peel—ellagic acid, punicalagins, flavonoids (e.g., quercetin), β-carotene, vitamin C Bagasse powder—ferulic acid, caffeic acid gallic acid, syringic acid, p-coumaric acid, vanillic acid, quercetin, rutin, kampferol, luteolin, β-carotene, lycopene, vitamin C	Pomegranate peel powder (2 g), pomegranate aril bagasse powder (4 g), pomegranate peel powder aqueous extract (6 g) and pomegranate aril bagasse powder aqueous extract (9 g)	Effect on quality characteristics	The treatments can be effectively used as a replacement of synthetic antioxidants	[134,135]
	Patties	*Prunus salicina* peel and pulp microparticles	Chlorogenic acid, neochlorogenic acid, cryptochlorogenic acid, rutin, β-carotene, lutein, vitamin C	2.0% *w*/*w* level	Antioxidant activity	The treatment reduced TBARS formation in uncooked patties by 50% during 10-day storage at 4.0 °C.	[136]
		Grape dietary fibre	Polyphenols Resveratrol	0.5, 1, 1.5 and 2% grape antioxidant dietary fibre	Antioxidant activity	The extract improved the antioxidant stability and radical scavenging activity	[116]
	Cooked turkey meat	Peach skin powder	Chlorogenic acid, neochlorogenic acid, caffeic acid, gallic acid, ellagic acid, quercetin, β-carotene, lutein, vitamin C	0.5%, 1% and 0.01% BHA	Antioxidant activity	The increased concentration of peach skin powder had a better antioxidant effect on ground turkey. O’Henry peach skin powder prevented oxidation at the same levels as BHA.	[137]
	Cooked chicken breast meat	*Prunus mume* (Japanese apricot) methanolic extracts	Phenolic acids Flavonoids Anthocyanins Vitamin C	Control, rosemary extract, 0.1%, *Prunus mume*, 0.1%	Antioxidant activity	TBARS values decreased with 45% in additional *Prunus mume* products compared with control	[138]

## Data Availability

Data is contained within the article.

## References

[1] FAO (1983). World Food Security: A Reappraisal of the Concepts and Approaches.

[2] Shah M.A., Bosco S.J.D., Mir S.A. (2014). Plant Extracts as Natural Antioxidants in Meat and Meat Products. Meat Sci..

[3] Caleja C., Barros L., Antonio A.L., Oliveira M.B.P.P., Ferreira I.C.F.R. (2017). A Comparative Study between Natural and Synthetic Antioxidants: Evaluation of Their Performance after Incorporation into Biscuits. Food Chem..

[4] Postali E., Peroukidou P., Giaouris E., Papachristoforou A. (2022). Investigating Possible Synergism in the Antioxidant and Antibacterial Actions of Honey and Propolis from the Greek Island of Samothrace through Their Combined Application. Foods.

[5] Pasqualone A., Bianco A.M., Paradiso V.M., Summo C., Gambacorta G., Caponio F., Blanco A. (2015). Production and Characterization of Functional Biscuits Obtained from Purple Wheat. Food Chem..

[6] Rasooli I. (2007). Food Preservation—A Biopreservative Approach. Food.

[7] Ye H., Shen S., Xu J., Lin S., Yuan Y., Jones G.S. (2013). Synergistic Interactions of Cinnamaldehyde in Combination with Carvacrol against Food-Borne Bacteria. Food Control.

[8] Karakaya M., Bayrak E., Ulusoy K. (2011). Use of Natural Antioxidants in Meat and Meat Products. J. Food Sci. Eng..

[9] Sawicki T., Starowicz M., Kłębukowska L., Hanus P. (2022). The Profile of Polyphenolic Compounds, Contents of Total Phenolics and Flavonoids, and Antioxidant and Antimicrobial Properties of Bee Products. Molecules.

[10] Rakasivi K.G.J., Chin K.B. (2022). Antioxidant Activity of Cinnamomum Cassia Extract and Quality of Raw Chicken Patties Added with C. Cassia Powder and Pleurotus Sajor-Caju Powder as Functional Ingredients during Storage. Anim. Biosci..

[11] Muzolf-Panek M., Kaczmarek A., Tomaszewska-Gras J., Cegielska-Radziejewska R., Majcher M. (2019). Oxidative and Microbiological Stability of Raw Ground Pork during Chilled Storage as Affected by Plant Extracts. Int. J. Food Prop..

[12] Efenberger-Szmechtyk M., Nowak A., Czyzowska A. (2021). Plant Extracts Rich in Polyphenols: Antibacterial Agents and Natural Preservatives for Meat and Meat Products. Crit. Rev. Food Sci. Nutr..

[13] Bajaj S., Urooj A., Prabhasankar P. (2006). Effect of Incorporation of Mint on Texture, Colour and Sensory Parameters of Biscuits. Int. J. Food Prop..

[14] Caleja C., Barros L., Antonio A.L., Ciric A., Soković M., Oliveira M.B.P.P., Santos-Buelga C., Ferreira I.C.F.R. (2015). *Foeniculum vulgare* Mill. as Natural Conservation Enhancer and Health Promoter by Incorporation in Cottage Cheese. J. Funct. Foods.

[15] Caleja C., Barros L., Antonio A.L., Ciric A., Barreira J.C.M., Sokovic´ M. (2015). Development of a Functional Dairy Food: Exploring Bioactive and Preservation Effects of Chamomile (*Matricaria recutita* L.). J. Funct. Foods.

[16] Reddy V., Urooj A., Kumar A. (2005). Evaluation of Antioxidant Activity of Some Plant Extracts and Their Application in Biscuits. Food Chem..

[17] Uzombah T.A. (2022). The Implications of Replacing Synthetic Antioxidants with Natural Ones in the Food Systems.

[18] Kumar Y., Yadav D.N., Ahmad T., Narsaiah K. (2015). Recent Trends in the Use of Natural Antioxidants for Meat and Meat Products. Compr. Rev. Food Sci. Food Saf..

[19] European Commission (2000). Commission Directive 2000/63/EC of 5 October 2000 amending Directive 96/77/EC laying down specific purity criteria on food additives other than colours and sweeteners. Off. J. L.

[20] European Commission (2000). Commission Directive 2001/30/EC of 2 May 2001 amending Directive 96/77/EC laying down specific purity criteria on food additives other than colours and sweeteners. Off. J. L.

[21] European Parliament, Council of the European Union (2002). Directive 2002/46/EC of the European Parliament and of the Council of 10 June 2002 on the approximation of the laws of the Member States relating to food supplements. Off. J. L.

[22] European Parliament, Council of the European Union (2004). Directive 2003/115/EC of the European Parliament and of the Council of 22 December 2003 amending Directive 94/35/EC on sweeteners for use in foodstuffs. Off. J. L.

[23] European Parliament, Council of the European Union (2009). Commission Directive 2009/32/EC of the European Parliament and of the Council of 23 April 2009 on the approximation of the laws of the Member States on extraction solvents used in the production of foodstuffs and food ingredients. Off. J. L.

[24] European Parliament, Council of the European Union (1995). European Parliament and Council Directive No 95/2/EC of 20 February 1995 on food additives other than colours and sweeteners. Off. J. L.

[25] European Parliament, Council of the European Union (1995). Commission Directive 95/31/EC as regards E 955 sucralose and E 962 salt of aspartame-acesulfame. Off. J. L.

[26] European Commission (1995). Commission Directive 95/45/EC of 26 July 1995 laying down specific purity criteria concerning colours for use in foodstuffs. Off. J. L.

[27] European Parliament, Council of the European Union (1996). Commission Directive 96/77/EC of 2 December 1996 laying down specific purity criteria on food additives other than colours and sweeteners. Off. J. L.

[28] European Parliament, Council of the European Union (1997). Directive 96/83/EC of the European Parliament and of the Council of 19 December 1996 amending Directive 94/35/EC on sweeteners for use in foodstuffs. Off. J. L.

[29] European Parliament, Council of the European Union (1997). Directive 96/85/EC of the European Parliament and of the Council of 19 December 1996 amending Directive 95/2/EC on food additives other than colours and sweeteners. Off. J. L.

[30] European Parliament, Council of the European Union (1998). Commission Directive 98/66/EC of 4 September 1998 amending Directive 95/31/EC laying down specific criteria of purity concerning sweeteners for use in foodstuffs. Off. J. L.

[31] European Parliament, Council of the European Union (2015). Directive (EU) 2015/2203 of the European Parliament and of the Council of 25 November 2015 on the approximation of the laws of the Member States relating to caseins and caseinates intended for human consumption and repealing Council Directive 83/417/EEC. Off. J. L.

[32] European Parliament, Council of the European Union (2004). Commission Directive 2004/46/EC of 16 April 2004 amending Directive 95/31/EC as regards E 955 sucralose and E 962 salt of aspartame-acesulfame. Off. J. L.

[33] European Parliament, Council of the European Union (2008). Regulation (EC) No 1333/2008 of the European Parliament and of the Council of 16 December 2008 on food additives. Off. J. L.

[34] Rather S.A., Masoodi F., Akhter R., Rather J.A., Shiekh K.A. (2016). Advances in Use of Natural Antioxidants as Food Additives for Improving the Oxidative Stability of Meat Products. Madr. J. Food Technol..

[35] Pokorný J. (2007). Are Natural Antioxidants Better-and Safer-than Synthetic Antioxidants?. Eur. J. Lipid Sci. Technol..

[36] Özcan M.M., Arslan D. (2011). Antioxidant Effect of Essential Oils of Rosemary, Clove and Cinnamon on Hazelnut and Poppy Oils. Food Chem..

[37] Sies H., Stahl W., Sundquist A.R. (1992). Antioxidant Functions of Vitamins. Vitamins E and C, Beta-Carotene, and Other Carotenoids. Ann. Acad. Sci..

[38] Serra V., Salvatori G., Pastorelli G. (2021). Dietary Polyphenol Supplementation in Food Producing Animals: Effects on the Quality of Derived Products. Animals.

[39] Lu W., Shi Y., Wang R., Su D., Tang M., Liu Y., Li Z. (2021). Antioxidant Activity and Healthy Benefits of Natural Pigments in Fruits: A Review. Int. J. Mol. Sci..

[40] Cazzonelli C.I., Pogson B.J. (2010). Source to Sink: Regulation of Carotenoid Biosynthesis in Plants. Trends Plant Sci..

[41] Luo H., He W., Dai Z., Zhang Z., Bao Y., Li D., Zhu P. (2022). Concurrent Production of α- and β-Carotenes with Different Stoichiometries Displaying Diverse Antioxidative Activities via Lycopene Cyclases-Based Rational System. Antioxidants.

[42] Black H.S., Boehm F., Edge R., Truscott T.G. (2020). The Benefits and Risks of Certain Dietary Carotenoids That Exhibit Both Anti- and Pro-Oxidative Mechanisms-A Comprehensive Review. Antioxidants.

[43] Cetinkaya H., Kulak M., Karaman M., Karaman H.S., Kocer F., Cetinkaya H., Kulak M., Karaman M., Karaman H.S., Kocer F. (2017). Flavonoid Accumulation Behavior in Response to the Abiotic Stress: Can a Uniform Mechanism Be Illustrated for All Plants?.

[44] Drappier J., Thibon C., Rabot A., Geny-Denis L. (2019). Relationship between Wine Composition and Temperature: Impact on Bordeaux Wine Typicity in the Context of Global Warming-Review. Crit. Rev. Food Sci. Nutr..

[45] Bourzeix M. (1993). Influence des proanthocyanidols du raisin et du vin sur la sante. Polyphenolic Phenomena.

[46] Bourzeix M., Weyland D., Heredia N. (1986). A study of catechins and procyanidins of grape clusters, the wine and other by-products of the wine. 1. Physiological interest, chemical nature of catechins and procyanidins; 2. Research works, [high performance liquid chromatography; grape jelly. Bull. De L’oiv.

[47] Mahboubi A., Asgarpanah J., Sadaghiyani P.N., Faizi M. (2015). Total Phenolic and Flavonoid Content and Antibacterial Activity of Punica Granatum, L. Var. Pleniflora Flowers (Golnar) against Bacterial Strains Causing Foodborne Diseases. BMC Complement. Altern. Med..

[48] Hashemi M., Ehsani A., Afshari A., Aminzare M., Raeisi M. (2016). Chemical Composition and Antifungal Effect of Echinophora Platyloba Essential Oil against Aspergillus Flavus, Penicillium Expansum and Fusarium Graminearum. J. Chem. Health Risks.

[49] Pisoschi A.M., Pop A., Iordache F., Stanca L., Predoi G., Serban A.I. (2021). Oxidative Stress Mitigation by Antioxidants-An Overview on Their Chemistry and Influences on Health Status. Eur. J. Med. Chem..

[50] Ajilaa K.C.M., LeelaVathib U.J.S. (2008). Prasada Raoa Improvement of Dietary Fiber Content and Antioxidant Properties in Soft Dough Biscuits with the Incorporation of Mango Peel Powder. J. Cereal Sci..

[51] Virot M., Tomao V., Le Bourvellec C., Renard C.M.C.G., Chemat F. (2010). Towards the Industrial Production of Antioxidants from Food Processing By-Products with Ultrasound-Assisted Extraction. Ultrason. Sonochemistry.

[52] Nissen L.R., Byrne D.V., Bertelsen G., Skibsted L.H. (2004). The Antioxidative Activity of Plant Extracts in Cooked Pork Patties as Evaluated by Descriptive Sensory Profiling and Chemical Analysis. Meat Sci..

[53] Sebranek J.G., Sewalt V.J.H., Robbins K.L., Houser T.A. (2005). Comparison of a Natural Rosemary Extract and BHA/BHT for Relative Antioxidant Effectiveness in Pork Sausage. Meat Sci..

[54] Mathenjwa S.A., Hugo C.J., Bothma C., Hugo A. (2012). Effect of Alternative Preservatives on the Microbial Quality, Lipid Stability and Sensory Evaluation of Boerewors. Meat Sci..

[55] Bañón S., Díaz P., Rodríguez M., Garrido M.D., Price A. (2007). Ascorbate, Green Tea and Grape Seed Extracts Increase the Shelf Life of Low Sulphite Beef Patties. Meat Sci..

[56] Colindres P., Brewer M.S. (2011). Oxidative Stability of Cooked, Frozen, Reheated Beef Patties: Effect of Antioxidants. J. Sci. Food Agric..

[57] Hayes J.E., Stepanyan V., Allen P., O’Grady M.N., Kerry J.P. (2011). Evaluation of the Effects of Selected Plant-Derived Nutraceuticals on the Quality and Shelf-Life Stability of Raw and Cooked Pork Sausages. LWT-Food Sci. Technol..

[58] Efenberger-Szmechtyk M., Gałązka-Czarnecka I., Otlewska A., Czyżowska A., Nowak A. (2021). Aronia Melanocarpa (Michx.) Elliot, Chaenomeles Superba Lindl. and Cornus Mas, L. Leaf Extracts as Natural Preservatives for Pork Meat Products. Molecules.

[59] Olivas-Méndez P., Chávez-Martínez A., Santellano-Estrada E., Guerrero Asorey L., Sánchez-Vega R., Rentería-Monterrubio A.L., Chávez-Flores D., Tirado-Gallegos J.M., Méndez-Zamora G. (2022). Antioxidant and Antimicrobial Activity of Rosemary (*Rosmarinus officinalis*) and Garlic (*Allium sativum*) Essential Oils and Chipotle Pepper Oleoresin (*Capsicum annum*) on Beef Hamburgers. Foods.

[60] Sayed Mostafa H., Fawzy El Azab E. (2022). Efficacy of Green Coffee as an Antioxidant in Beef Meatballs Compared with Ascorbic Acid. Food Chem. X.

[61] Biasi V., Huber E., Goldoni T.S.H., de Melo A.P.Z., Hoff R.B., Verruck S., Barreto P.L.M. (2023). Goldenberry Flour as a Natural Antioxidant in Bologna-Type Mortadella during Refrigerated Storage and in Vitro Digestion. Meat Sci..

[62] Pokorný J., Yanishlieva N., Gordon M. Antioxidants in Food 1st Ed. https://www.elsevier.com/books/antioxidants-in-food/pokorny/978-1-85573-463-0.

[63] Manessis G., Kalogianni A.I., Lazou T., Moschovas M., Bossis I., Gelasakis A.I. (2020). Plant-Derived Natural Antioxidants in Meat and Meat Products. Antioxidants.

[64] European Parliament and of the Council (2003). Regulation (EC) No 1831/2003 of the European Parliament and of the Council of 22 September 2003 on Additives for Use in Animal Nutrition (Text with EEA Relevance). Off. J. Eur. Union.

[65] 21 CFR 182.20-Essential Oils, Oleoresins (Solvent-Free), and Natural Extractives (Including Distillates). https://www.ecfr.gov/current/title-21/chapter-I/subchapter-B/part-182/subpart-A/section-182.20.

[66] 21 CFR 182.10-Spices and Other Natural Seasonings and Flavorings. https://www.ecfr.gov/current/title-21/chapter-I/subchapter-B/part-182/subpart-A/section-182.10.

[67] Canada H. 11. List of Permitted Preservatives (Lists of Permitted Food Additives). https://www.canada.ca/en/health-canada/services/food-nutrition/food-safety/food-additives/lists-permitted/11-preservatives.html.

[68] Huang X., Ahn D.U. (2019). Lipid Oxidation and Its Implications to Meat Quality and Human Health. Food Sci. Biotechnol..

[69] Domínguez R., Pateiro M., Gagaoua M., Barba F.J., Zhang W., Lorenzo J.M. (2019). A Comprehensive Review on Lipid Oxidation in Meat and Meat Products. Antioxidants.

[70] Tian F., Decker E.A., Goddard J.M. (2013). Controlling Lipid Oxidation of Food by Active Packaging Technologies. Food Funct.

[71] Ahna J., Grun I.U., Mustaphab A. (2007). Effects of Plant Extracts on Microbial Growth, Color Change, and Lipid Oxidation in Cooked Beef. Food Microbiol..

[72] Yin H., Xu L., Porter N.A. (2011). Free Radical Lipid Peroxidation: Mechanisms and Analysis. Chem. Rev..

[73] Aminzare M., Hashemi M., Ansarian E., Bimkar M., Azar H.H., Mehrasbi M.R., Daneshamooz S., Raeisi M., Behrooz J. (2019). Asma A Using Natural Antioxidants in Meat and Meat Products as Preservatives: A Review. Adv. Anim. Vet. Sci..

[74] MacDonald-Wicks L.K., Wood L.G., Garg M.L. (2006). Methodology for the Determination of Biological Antioxidant Capacity in Vitro: A Review. J. Sci. Food Agric..

[75] Santos-Sánchez N.F., Salas-Coronado R., Villanueva-Cañongo C., Hernández-Carlos B., Santos-Sánchez N.F., Salas-Coronado R., Villanueva-Cañongo C., Hernández-Carlos B. (2019). Antioxidant Compounds and Their Antioxidant Mechanism.

[76] Ponnampalam E.N., Kiani A., Santhiravel S., Holman B.W.B., Lauridsen C., Dunshea F.R. (2022). The Importance of Dietary Antioxidants on Oxidative Stress, Meat and Milk Production, and Their Preservative Aspects in Farm Animals: Antioxidant Action, Animal Health, and Product Quality—Invited Review. Animals.

[77] Surai P.F. (2014). Polyphenol Compounds in the Chicken/Animal Diet: From the Past to the Future. J. Anim. Physiol. Anim. Nutr..

[78] Yañez O., Osorio M.I., Osorio E., Tiznado W., Ruíz L., García C., Nagles O., Simirgiotis M.J., Castañeta G., Areche C. (2023). Antioxidant Activity and Enzymatic of Lichen Substances: A Study Based on Cyclic Voltammetry and Theoretical. Chem. Biol. Interact..

[79] White P.A.S., Oliveira R.C.M., Oliveira A.P., Serafini M.R., Araújo A.A.S., Gelain D.P., Moreira J.C.F., Almeida J.R.G.S., Quintans J.S.S., Quintans-Junior L.J. (2014). Antioxidant Activity and Mechanisms of Action of Natural Compounds Isolated from Lichens: A Systematic Review. Molecules.

[80] McCord J.M. (2000). The Evolution of Free Radicals and Oxidative Stress. Am. J. Med..

[81] Trautwein C., Friedman S.L., Schuppan D., Pinzani M. (2015). Hepatic Fibrosis: Concept to Treatment. J. Hepatol..

[82] Banerjee R., Verma A.K., Siddiqui M.W. (2017). Applications in Foods of Animal Origin.

[83] Noguchi N., Watanabe A., Shi H. (2000). Diverse Functions of Antioxidants. Free Radic. Res..

[84] Ighodaro O.M., Akinloye O.A. (2018). First Line Defence Antioxidants-Superoxide Dismutase (SOD), Catalase (CAT) and Glutathione Peroxidase (GPX): Their Fundamental Role in the Entire Antioxidant Defence Grid. Alex. J. Med..

[85] Efenberger-Szmechtyk M., Nowak A., Czyżowska A., Śniadowska M., Otlewska A., Żyżelewicz D. (2021). Antibacterial Mechanisms of Aronia Melanocarpa (Michx.), Chaenomeles Superba Lindl. and Cornus Mas, L. Leaf Extracts. Food Chem..

[86] Branciari R., Galarini R., Giusepponi D., Trabalza-Marinucci M., Forte C., Roila R., Miraglia D., Servili M., Acuti G., Valiani A. (2017). Oxidative Status and Presence of Bioactive Compounds in Meat from Chickens Fed Polyphenols Extracted from Olive Oil Industry Waste. Sustainability.

[87] Animals Free Full-Text Red Grape Pomace Rich in Polyphenols Diet Increases the Antioxidant Status in Key Organs—Kidneys, Liver, and Spleen of Piglets. https://www.mdpi.com/2076-2615/9/4/149.

[88] Kafantaris I., Kotsampasi B., Christodoulou V., Makri S., Stagos D., Gerasopoulos K., Petrotos K., Goulas P., Kouretas D. (2018). Effects of Dietary Grape Pomace Supplementation on Performance, Carcass Traits and Meat Quality of Lambs. In Vivo.

[89] Hernández P., Zomeño L., Ariño B., Blascoinvivo A. (2004). Antioxidant, Lipolytic and Proteolytic Enzyme Activities in Pork Meat from Different Genotypes. Meat Sci..

[90] Velasco V., Williams Salinas P. (2011). Improving Meat Quality through Natural Antioxidants. Chil. J. Agric. Res..

[91] Castillo C., Pereira V., Abuelo Á., Hernández J. (2013). Effect of Supplementation with Antioxidants on the Quality of Bovine Milk and Meat Production. Sci. World J..

[92] Descalzo A.M., Insani E.M., Biolatto A., Sancho A.M., García P.T., Pensel N.A., Josifovich J.A. (2005). Influence of Pasture or Grain-Based Diets Supplemented with Vitamin E on Antioxidant/Oxidative Balance of Argentine Beef. Meat Sci..

[93] Guo Q., Richert B.T., Burgess J.R., Webel D.M., Orr D.E., Blair M., Grant A.L., Gerrard D.E. (2006). Effect of Dietary Vitamin E Supplementation and Feeding Period on Pork Quality. J. Anim. Sci..

[94] Onibi G.E., Scaife J.R., Murray I., Fowler V.R. (2000). Supplementary α-Tocopherol Acetate in Full-Fat Rapeseed-Based Diets for Pigs: Influence on Tissue α-Tocopherol Content, Fatty Acid Profiles and Lipid Oxidation. J. Sci. Food Agric..

[95] Rossi R., Pastorelli G., Cannata S., Tavaniello S., Maiorano G., Corino C. (2013). Effect of Long Term Dietary Supplementation with Plant Extract on Carcass Characteristics Meat Quality and Oxidative Stability in Pork. Meat Sci..

[96] Norkeaw R., Arjin C., Sartsook A., Hnokaew P., Thongkham M., Detruengsri B., Chaiwang N., Mekchay S., Yano T., Sringarm K. (2022). Effect of Dietary Green Tea Extract Supplementation in Growing-Finishing Pigs on Growth Performance, Meat Quality, and Oxidative Stability of Pork. Vet. Integr. Sci..

[97] O’Grady M.N., Carpenter R., Lynch P.B., O’Brien N.M., Kerry J.P. (2008). Addition of Grape Seed Extract and Bearberry to Porcine Diets: Influence on Quality Attributes of Raw and Cooked Pork. Meat Sci..

[98] Hernández-López S.H., Rodríguez-Carpena J.G., Lemus-Flores C., Galindo-García J., Estévez M. (2016). Antioxidant Protection of Proteins and Lipids in Processed Pork Loin Chops through Feed Supplementation with Avocado. J. Food Sci. Technol..

[99] Chedea V.S., Palade L.M., Marin D.E., Pelmus R.S., Habeanu M., Rotar M.C., Gras M.A., Pistol G.C., Taranu I. (2018). Intestinal Absorption and Antioxidant Activity of Grape Pomace Polyphenols. Nutrients.

[100] Yi Z., QuanHang X., Jun W., HongKui W., Jian P. (2016). Effects of Oregano Essential Oil or Quercetin Supplementation on Body Weight Loss, Carcass Characteristics, Meat Quality and Antioxidant Status in Finishing Pigs under Transport Stress. Livest. Sci..

[101] Faustman C., Chan W.K., Schaefer D.M., Havens A. (1998). Beef Color Update: The Role for Vitamin, E. J. Anim. Sci..

[102] Galvin K., Lynch A.M., Kerry J.P., Morrissey P.A., Buckley D.J. (2000). Effect of Dietary Vitamin E Supplementation on Cholesterol Oxidation in Vacuum Packaged Cooked Beef Steaks. Meat Sci..

[103] Liu Q., Scheller K.K., Schaefer D.M., Arp S.C., Williams S.N. (1994). Dietary α-Tocopheryl Acetate Contributes to Lipid Stability in Cooked Beef. J. Food Sci..

[104] Faustman C., Cassens R.G., Schaefer D.M., Buege D.R., Williams S.N., Scheller K.K. (1989). Improvement of Pigment and Lipid Stability in Holstein Steer Beef by Dietary Supplementation with Vitamin, E. J. Food Sci..

[105] Mercier Y., Gatellier P., Renerre M. (2004). Lipid and Protein Oxidation in Vitro, and Antioxidant Potential in Meat from Charolais Cows Finished on Pasture or Mixed Diet. Meat Sci..

[106] Vlahova-Vangelova D.B., Balev D.K., Kolev N.D., Terziyska M.N., Dragoev S.G. (2021). The Effect of Dietary Dry Distilled Rose Petals or Dihydroquercetin Supplementation on the Oxidative Stability and Quality of Lamb Muscles and Fat. Agrirxiv.

[107] Daley C.A., Abbott A., Doyle P.S., Nader G.A., Larson S. (2010). A Review of Fatty Acid Profiles and Antioxidant Content in Grass-Fed and Grain-Fed Beef. Nutr. J..

[108] Luciano G., Roscini V., Mattioli S., Ruggeri S., Gravador R.S., Natalello A., Lanza M., De Angelis A., Priolo A. (2017). Vitamin E Is the Major Contributor to the Antioxidant Capacity in Lambs Fed Whole Dried Citrus Pulp. Animal.

[109] Gómez-Cortés P., Guerra-Rivas C., Gallardo B., Lavín P., Mantecón A.R., de la Fuente M.A., Manso T. (2018). Grape Pomace in Ewes Diet: Effects on Meat Quality and the Fatty Acid Profile of Their Suckling Lambs. Food Res. Int..

[110] Yagoubi Y., Joy M., Ripoll G., Mahouachi M., Bertolín J.R., Atti N. (2018). Rosemary Distillation Residues Reduce Lipid Oxidation, Increase Alpha-Tocopherol Content and Improve Fatty Acid Profile of Lamb Meat. Meat Sci..

[111] Gobert M., Gruffat D., Habeanu M., Parafita E., Bauchart D., Durand D. (2010). Plant Extracts Combined with Vitamin E in PUFA-Rich Diets of Cull Cows Protect Processed Beef against Lipid Oxidation. Meat Sci..

[112] Karami M., Ponnampalam E.N., Hopkins D.L. (2013). The Effect of Palm Oil or Canola Oil on Feedlot Performance, Plasma and Tissue Fatty Acid Profile and Meat Quality in Goats. Meat Sci..

[113] Munekata P.E.S., Gullón B., Pateiro M., Tomasevic I., Domínguez R., Lorenzo J.M. (2020). Natural Antioxidants from Seeds and Their Application in Meat Products. Antioxidants.

[114] Fellenberg M.A., Speisky H. (2006). Antioxidants: Their Effects on Broiler Oxidative Stress and Its Meat Oxidative Stability. World’s Poult. Sci. J..

[115] Avila-Ramos F., Pro-Martínez A., Sosa-Montes E., Cuca-García J.M., Becerril-Pérez C., Figueroa-Velasco J.L., Ruiz-Feria C.A., Hernández-Cázares A.S., Narciso-Gaytán C. (2013). Dietary Supplemented and Meat-Added Antioxidants Effect on the Lipid Oxidative Stability of Refrigerated and Frozen Cooked Chicken Meat. Poult. Sci..

[116] Sáyago-Ayerdi S.G., Brenes A., Viveros A., Goñi I. (2009). Antioxidative Effect of Dietary Grape Pomace Concentrate on Lipid Oxidation of Chilled and Long-Term Frozen Stored Chicken Patties. Meat Sci..

[117] Vieira V., Marx F.O., Bassi L.S., Santos M.C., Oba A., de Oliveira S.G., Maiorka A. (2021). Effect of Age and Different Doses of Dietary Vitamin E on Breast Meat Qualitative Characteristics of Finishing Broilers. Anim. Nutr..

[118] Aditya S., Ohh S.-J., Ahammed M., Lohakare J. (2018). Supplementation of Grape Pomace (Vitis Vinifera) in Broiler Diets and Its Effect on Growth Performance, Apparent Total Tract Digestibility of Nutrients, Blood Profile, and Meat Quality. Anim. Nutr..

[119] Tufarelli V., Laudadio V., Casalino E. (2016). An Extra-Virgin Olive Oil Rich in Polyphenolic Compounds Has Antioxidant Effects in Meat-Type Broiler Chickens. Env. Sci. Pollut. Res. Int..

[120] Roila R., Valiani A., Miraglia D., Ranucci D., Forte C., Trabalza-Marinucci M., Servili M., Codini M., Branciari R. (2018). Olive Mill Wastewater Phenolic Concentrate as Natural Antioxidant against Lipid-Protein Oxidative Deterioration in Chicken Meat during Storage. Ital. J. Food Saf..

[121] Gumus R., Gelen S.U. (2023). Effects of Dietary Thyme and Rosemary Essential Oils on Performance Parameters with Lipid Oxidation, Water Activity, PH, Colour and Microbial Quality of Breast and Drumstick Meats in Broiler Chickens. Arch. Anim. Breed..

[122] Calderón-Oliver M., López-Hernández L.H. (2022). Food Vegetable and Fruit Waste Used in Meat Products. Food Rev. Int..

[123] Cunha L.C.M., Monteiro M.L.G., Lorenzo J.M., Munekata P.E.S., Muchenje V., de Carvalho F.A.L., Conte-Junior C.A. (2018). Natural Antioxidants in Processing and Storage Stability of Sheep and Goat Meat Products. Food Res. Int..

[124] Kalogianni A.I., Lazou T., Bossis I., Gelasakis A.I. (2020). Natural Phenolic Compounds for the Control of Oxidation, Bacterial Spoilage, and Foodborne Pathogens in Meat. Foods.

[125] Pateiro M., Lorenzo J.M., Amado I.R., Franco D. (2014). Effect of Addition of Green Tea, Chestnut and Grape Extract on the Shelf-Life of Pig Liver Pâté. Food Chem..

[126] Qin Y.-Y., Zhang Z.-H., Li L., Xiong W., Shi J.-Y., Zhao T.-R., Fan J. (2013). Antioxidant Effect of Pomegranate Rind Powder Extract, Pomegranate Juice, and Pomegranate Seed Powder Extract as Antioxidants in Raw Ground Pork Meat. Food Sci. Biotechnol..

[127] Rodrigues A.S., Kubota E.H., da Silva C.G., Dos Santos Alves J., Hautrive T.P., Rodrigues G.S., Campagnol P.C.B. (2020). Banana Inflorescences: A Cheap Raw Material with Great Potential to Be Used as a Natural Antioxidant in Meat Products. Meat Sci..

[128] Nuñez de Gonzalez M.T., Hafley B.S., Boleman R.M., Miller R.M., Rhee K.S., Keeton J.T. (2009). Qualitative Effects of Fresh and Dried Plum Ingredients on Vacuum-Packaged, Sliced Hams. Meat Sci..

[129] Garrido M.D., Auqui M., Martí N., Linares M.B. (2011). Effect of Two Different Red Grape Pomace Extracts Obtained under Different Extraction Systems on Meat Quality of Pork Burgers. LWT-Food Sci. Technol..

[130] García-Lomillo J., Gonzalez-SanJose M.L., Pino-García R.D., Ortega-Heras M., Muñiz-Rodríguez P. (2017). 1 Antioxidant Effect of Seasonings Derived from Wine Pomace in 2 Refrigerated and Frozen Beef Patties. LWT.

[131] Rojas M.C., Brewer M.S. (2008). Effect of Natural Antioxidants on Oxidative Stability of Frozen, Vacuum-Packaged Beef and Pork. J. Food Qual..

[132] Morsy M.K., Mekawi E., Elsabagh R. (2018). Impact of Pomegranate Peel Nanoparticles on Quality Attributes of Meatballs during Refrigerated Storage. LWT.

[133] Kulkarni S., DeSantos F.A., Kattamuri S., Rossi S.J., Brewer M.S. (2011). Effect of Grape Seed Extract on Oxidative, Color and Sensory Stability of a Pre-Cooked, Frozen, Re-Heated Beef Sausage Model System. Meat Sci..

[134] Sharma P., Yadav S. (2020). Effect of Incorporation of Pomegranate Peel and Bagasse Powder and Their Extracts on Quality Characteristics of Chicken Meat Patties. Food Sci. Anim. Resour..

[135] Sharma H., Mendiratta S.K., Agarwal R.K., Kumar S., Soni A. (2017). Evaluation of Anti-Oxidant and Anti-Microbial Activity of Various Essential Oils in Fresh Chicken Sausages. J. Food Sci. Technol..

[136] Basanta M.F., Rizzo S.A., Szerman N., Vaudagna S.R., Descalzo A.M., Gerschenson L.N., Pérez C.D., Rojas A.M. (2018). Plum (Prunus Salicina) Peel and Pulp Microparticles as Natural Antioxidant Additives in Breast Chicken Patties. Food Res. Int..

[137] Zhang Y., Han I., Bridges W.C., Dawson P.L. (2016). Peach Skin Powder Inhibits Oxidation in Cooked Turkey Meat. Poult. Sci..

[138] Jo S.-C., Nam K.-C., Min B.-R., Ahn D.-U., Cho S.-H., Park W.-P., Lee S.-C. (2006). Antioxidant Activity of Prunus Mume Extract in Cooked Chicken Breast Meat. Int. J. Food Sci. Technol..

[139] Oswell N.J., Thippareddi H., Pegg R.B. (2018). Practical Use of Natural Antioxidants in Meat Products in the U.S.: A Review. Meat Sci..

[140] Jayasena D.D., Jo C. (2014). Potential Application of Essential Oils as Natural Antioxidants in Meat and Meat Products: A Review. Food Rev. Int..

[141] Estévez M. (2021). Critical Overview of the Use of Plant Antioxidants in the Meat Industry: Opportunities, Innovative Applications and Future Perspectives. Meat Sci..

[142] Castañeda-Arriaga R., Pérez-González A., Reina M., Alvarez-Idaboy J.R., Galano A. (2018). Comprehensive Investigation of the Antioxidant and Pro-Oxidant Effects of Phenolic Compounds: A Double-Edged Sword in the Context of Oxidative Stress?. J. Phys. Chem. B.

[143] European Parliament, Council of the European Union (2018). Commission Regulation (EU) 2018/1481 of 4 October 2018 Amending Annexes II and III to Regulation (EC) No 1333/2008 of the European Parliament and of the Council and the Annex to Commission Regulation (EU) No 231/2012 as Regards Octyl Gallate (E 311) and Dodecyl Gallate (E 312) (Text with EEA Relevance.). Off. J. L.

[144] CFR—Code of Federal Regulations Title 21. https://www.accessdata.fda.gov/scripts/cdrh/cfdocs/cfcfr/CFRSearch.cfm?CFRPart=172.

[145] Santamaria P. (2006). Nitrate in Vegetables: Toxicity, Content, Intake and EC Regulation. J. Sci. Food Agric..

[146] Soladoye O.P., Juárez M.L., Aalhus J.L., Shand P., Estévez M. (2015). Protein Oxidation in Processed Meat: Mechanisms and Potential Implications on Human Health. Compr. Rev. Food Sci. Food Saf..

